# What do teachers think about students’ emotions? A mixed-method exploration of their implicit theories

**DOI:** 10.1007/s10212-026-01074-5

**Published:** 2026-03-05

**Authors:** Line Fischer, Catherine Audrin, Sandrine Biémar, Nathalie Mella, David Sander, Marc Romainville, Elise Dan-Glauser

**Affiliations:** 1 FASEF, University of Namur, Rue de Bruxelles 61, B-5000 Namur, Belgium; 2 Haute Ecole Pédagogique Vaud, Av. de Cour 33, Lausanne, Switzerland; 3 FPSE, University of Geneva, Uni Mail, 40, Boulevard du Pont-d’Arve, 1211 Geneva 4, Switzerland; 4 Swiss Center for Affective Sciences, University of Geneva, Campus Biotech, 9 Chemin Des Mines, 1202 Geneva, Switzerland; 5 Institute of Psychology, Unil-Mouline, University of Lausanne, 1015 Lausanne, Switzerland

**Keywords:** Teachers’ beliefs, Students’ emotions, Utility of emotion, Source of emotion, Malleability of emotion, Implicit theories of emotions

## Abstract

Emotions play a central role in educational settings, influencing both students’ engagement and teachers’ instructional practices. Despite growing recognition of the importance of emotion in education, little is known so far about how preservice teachers conceptualize emotions in students and how this may shape their pedagogical choices. This mixed-method study explored the beliefs of 292 Belgian and Swiss teachers in training about students' emotions, knowing that these emotional beliefs are crucial in influencing teaching practices, interactions and students’ outcomes. Results showed that teachers attributed positive emotions such as interest and pride more to their own influence, whereas emotions such as joy and shame as externally driven. They saw emotions as variably useful depending on context: interest, pride, and joy being particularly useful for learning. Finally, beliefs in emotion malleability were linked to perceived utility only when aiming at enhancing relationships with the teachers. Findings emphasize the need to integrate emotional education in teacher training to provide scientific knowledge of the role of emotions in learning and teaching. Such integration could foster emotionally responsive teaching practices and enhance both teacher well-being and student success.

## Introduction

It is well-established that emotions shape several key aspects of students’ functioning in the classroom, including their motivation (Boekaerts, 2010) and behavioral engagement, which refers to the effort and persistence in learning tasks (Pekrun & Linnenbrink-Garcia, 2012). More specifically, emotions also affect cognition and, in particular, core learning processes. Indeed, by enhancing or hindering attention, memory, and problem-solving, emotions directly affect the students’ learning processes, i.e., the mechanism by which one absorbs and retains information (Brosch et al., 2013; Denervaud et al., 2017) .

All individuals hold beliefs about emotions, specifically their origins, effects, and how they could be managed (Ford & Gross, 2019; Peter et al., 2025). These beliefs, however, are not always grounded in reality; they may be shaped by cultural myths (e.g., certain emotions are more desirable than others or are better expressed in certain contexts, Boiger & Mesquita, 2012) or personal misconceptions (e.g., an unpleasant emotion is inevitably negative for the individual, De Castella et al., 2013) that do not necessarily reflect the nature of emotions and their impact on learning.

Given the critical role that teachers play in shaping the educational experiences of their students, understanding teachers’ beliefs about the role of emotions in learning becomes essential but, so far, scarce research has investigated such beliefs. Since teachers’ perceptions on students’ emotions shape their instructional practices, classroom management, and interactions, it is essential to examine their beliefs about emotions in learning. Addressing these beliefs helps to optimize educational outcomes and use emotions more effectively in the classroom.

### Emotions and their impact on learning

Emotions are complex phenomena involving subjective feelings, physiological changes, and behavioral responses (Scherer, 2005). They are triggered by significant events occurring in the individuals’ environment and serve adaptive functions (Adolphs, 2010). Emotions and cognitive processes are intrinsically linked as they involve overlapping neural systems and bottom-up and top-down mechanisms (Dolcos & Denkova, 2014). Emotions hence play a significant and multifaceted role in learning processes, by profoundly influencing various cognitive functions that are crucial for effective learning (Gkintoni et al., 2023; Tyng et al., 2017), such as attention mechanism and problem-solving skills. Both positive and negative emotional states have the capacity to impact learning outcomes (Camacho-Morles et al., 2021), with positive emotions generally enhancing performance and engagement (Córdova et al., 2023). Indeed, epistemic emotions like surprise and curiosity interact with metacognition, influencing self-regulated learning (Efklides, 2017; Muis et al., 2018). Negative emotions have often been viewed as hindering or disrupting the learning experience (Córdova et al., 2023; Pekrun, 1992), but emotions such as anger, sadness, fear, and boredom can be beneficial under certain circumstances (Rowe & Fitness, 2018). Indeed, studies have shown that negative emotions can enhance motivation, cognitive reflection, and learning outcomes (Knörzer et al., 2016; Matsunobu et al., 2022). Emotions can thus significantly either facilitate or hinder the learning process by impacting variables that foster learning, such as students’ motivation and engagement (Garcia Retana, 2012; LeBlanc & Posner, 2022).

Hence, educators and educational researchers must consider the emotional experiences of students and implement strategies that harness the potential of positive emotions to enhance learning, while mitigating the detrimental effects of negative emotions. To do so, they need to have a reliable perception of the utility, source, and malleability of students’ emotions, which might differ from their lay representations.

### Beliefs about utility, source, and malleability of emotions

Research suggests that individuals hold a variety of beliefs about the nature and dynamics of emotions, which significantly shape their emotional experiences and behaviors. These beliefs encompass several dimensions, such as whether emotions are inherently useful, whether they can be controlled or are beyond one’s control, and which are their sources (Ford & Gross, 2019).

These foundational views about emotions may influence how teachers experience, regulate, and respond to their own and those of their students. For instance, teachers who view emotions as malleable (which means capable of change) are more likely to engage in active and effortful regulation strategies when facing emotionally challenging classroom situations (De Castella et al., 2013). This proactive approach to emotion regulation should lead to more adaptive emotional outcomes and greater resilience in the face of daily teaching demands (Kneeland & Dovidio, 2020; Tabibnia & Radecki, 2018). Moreover, teachers’ implicit theories about emotions, particularly beliefs about their malleability, should play an important role in shaping classroom relationships (Ford & Gross, 2018). Those who perceive students’ emotions as malleable may be better equipped to navigate complex social dynamics in the classroom, and, thus, to respond more constructively to students’ emotional expressions and to manage their own emotional reactions more effectively, supporting a more positive and responsive classroom climate.

Understanding the diverse beliefs teachers hold about the nature, utility and malleability of emotions is therefore essential, as these beliefs not only shape teachers’ emotional functioning but also influence the emotional and relational dynamics within their classroom. Research, however, suggests that people's beliefs about psychological processes often do not align with the way the mind works as currently described by actual research. This possible mismatch between subjective beliefs and scientific knowledge can occur in a wide range of contexts (i.e., the recollection of autobiographical memories, Blank, 2017; or the reasoning about the thoughts and intentions of others, Liu et al., 2004).

Regarding the educational domain, Hanson-Peterson et al. (2016, p. 16) note that “Despite the recognized influence of teachers on their students’ social and emotional development, there remains a paucity of research examining teachers’ emotion beliefs […].” They add that most of the research to date has focused on the beliefs of early childhood educators, in particular about whether and how early childcare teachers should teach children emotional expression (Hyson & Lee, 1996). Hagan et al. (2020) highlight that research is needed on teachers’ beliefs about which students’ emotions they consider useful or not for learning (and not just which emotions they consider appropriate or inappropriate to express). Recent studies are beginning to follow this path with regard to specific emotions (such as anger, Hagan et al., 2020). This research therefore aims to address this gap identified in the literature, in particular by opening up the exploration to a wider range of emotions.

### Impact of teachers’ beliefs on their practice and learning outcome

Research indicates that teachers’ beliefs significantly influence the teaching–learning process, affecting their instructional strategies, classroom practices, and student outcomes (Solis, 2015; Xu, 2012). Teachers’ beliefs about the development of emotion socialization influence their approach to emotional competence development in students (Morris et al., 2013) and their implementation of social-emotional learning programs (Hanson, 2012). This suggests that emotional beliefs are not peripheral, but central to how teachers shape students’ socio-emotional experiences. These beliefs are interconnected with teachers’ own emotions (Barcelos & Ruohotie-Lyhty, 2018), affecting their support for students’ psychological needs (Jingwen et al., 2019) and classroom management strategies (Williams-Johnson et al., 2008). This is consistent with Pekrun’s Control-Value Theory of Achievement Emotions (Pekrun & Perry, 2014), which posits that students’ emotions are dependent on their perceived control over learning activities and on the subjective value they assign to them—both of which can be shaped by teachers’ feedback and instructional approaches. Thus, teachers’ beliefs about the sources of students’ emotions in the classroom, e.g., that they are partly shaped by instructional situations, appear important, as they might be aware that, to a certain extent, they are responsible for these emotional experiences. Ultimately, teachers’ beliefs about emotions play a significant role in shaping their instructional practices (Becker et al., 2014) and student relationships (Fives et al., 2019). Moreover, teachers’ implicit theories about emotions influence their behavior and thinking about children’s emotional development (Delaney, 1997). Often formed and shaped by culture or personal experience, they provide powerful yet underexplored frameworks that guide teachers’ expectations and interventions. Investigating these theories is essential for developing effective teacher education programs and improving classroom practices (Vosniadou, 2019).

### The present study

Past studies have provided some evidence that emotions’ beliefs influence emotional experiences and behaviors (e.g., De Castella et al., 2013; Ford & Gross, 2019), but this topic has only recently been explored among teachers. However, these studies mainly explored teachers’ beliefs about their own emotions and linked them to different outcomes (teachers’ well-being, professional commitment, teaching process, etc.). While important, this perspective overlooks the interpersonal dimension of teaching, where teachers constantly interpret and respond to students’ emotions. Existing work has not sufficiently addressed how teachers conceptualize students’ emotional experiences, nor how these beliefs may guide their pedagogical decisions or emotional support strategies in the classroom. We have therefore identified a critical gap in the existing literature, namely that studies have focused little on teachers’ beliefs about their students’ emotions and how teachers think they may impact learning processes.

To explore, quantitatively and qualitatively, the constellation of beliefs teachers may have about their students’ emotions, we conducted a mixed-method study in Switzerland and in the french-part of Belgium. We believe that the mixed-method design may provide interesting insights as it enables both the measurement of general patterns and a deeper exploration of participants’ perceptions and beliefs. Teachers in training were asked about their beliefs about emotion source, utility, and malleability. We focused on nine emotion categories (including pleasant and unpleasant emotions). Our approach was exploratory in nature, given the scarcity of research on the topic. We defined 5 research questions to guide this work:

- Q1: Is the perceived source of emotions felt in class differentially attributed (external factors vs. teacher-driven) depending on the specific emotion considered?
- Q2: Are emotions perceived as having different utility for learning processes or relationships in class? Do teachers evaluate differently the utility of such emotions depending on the utility domain, that is, whether they are seen as helpful for learning processes, student engagement, or performance?
- Q3: When teachers believe emotions can be changed, we think they could be more likely to see them as resources that can be regulated and used to support learning or relationships, thereby increasing their perceived utility. We hence question: Are beliefs in the malleability of students’ emotions associated with the perceived utilities?
- Q4: What beliefs do teachers have about the role of emotions in learning?
- Q5: What beliefs do teachers have about the role of emotions in the student–teacher relationship?

## Methods

### Participants

Teachers in training at the University of Namur, the University of Teacher Education of the Canton of Vaud (named HEP Vaud), the University of Lausanne, and the University of Geneva were invited to take part in our online survey. Our sample consisted of 292 respondents: 211 were women (72.3%), 79 were men (27.1%), and 2 students (0.7%) defined themselves as “non-binary.” One hundred eighty-seven respondents (64%) were between 18 and 25 years old, 53 (18.2%) between 26 and 35 years old, 44 (15.1%) between 36 and 50 years old, and 7 (2.4%) above 50 years old (1 participant, 0.3%, did not answer the age question). In our sample, 175 participants (59.9%) came from the University of Geneva, 62 (21.2%) from the University of Namur, 45 (15.4%) from HEP Vaud, and 4 (1.5%) from the University of Lausanne. The participation rates were 87% in the University of Geneva, 80% in the University of Namur, and 3% in HEP Vaud. We do not have data on the participation rates for the University of Lausanne sample.

Among the 292 respondents, 42.3% had previous teaching experience. 66.5% were in the first year of initial teacher training, 26.5% in the second year, and 6.5% in the third year. Thirty-four percent intend to teach in primary schools and 66% in secondary schools. 

Ethics approval for this project was granted by the University of Namur (n°2023/2) and the University of Geneva (n° CUREG-2023-05-63). All participants provided informed consents for their participation and for their data to be used.

### Procedure

All 292 participants completed the online survey in an anonymous way during a break course or at home. The questionnaire was sent to all students in teacher training at the University of Namur, in the first bachelor’s degree in teacher training at the University of Geneva and at the HEP Vaud. Its completion time was between 20 and 30 min. The questionnaire started with an informative part, followed by the informed consent section where participants agreed to be part of the study. Then, sociodemographic data were gathered, where we asked about the participant’s gender, age, the country in which they are training to be a teacher, the level of teaching they have chosen, and their past experience as a teacher. Three open questions were then asked about emotion definition, their role in learning, and their role in the relationship between teachers and students. Then, quantitative measures were gathered about their beliefs and their individual characteristics. No incentive was planned for participation.

### Measures

#### Quantitative measures of teachers’ beliefs about emotions

Whenever possible, different emotions were investigated. We selected nine main emotions including three positive (joy, interest, and pride) and six negative (anger, shame, frustration, anxiety, discouragement and boredom). It was important to have both positive and negative emotions, given the heightened influence of valence on the learning process (Megalakaki et al., 2019; Wortha et al., 2019). As in established instruments such as Pekrun’s AEQ (Pekrun et al., 2011), negative emotions were intentionally overrepresented because they display greater functional diversity and are more strongly associated with challenges in classroom learning. Moreover, we expected these particular emotions to both be felt frequently in a classroom (Bowen et al., 1988; Pekrun et al., 2002a, b) and to adequately portray a vast range of emotion types that are important in a teaching context, such as epistemic (e.g., boredom), achievement (e.g., anxiety), and social emotions such as shame (Sznycer et al., 2021; Vogl et al., 2019; Wilutzky, 2015).

- Beliefs about the source of emotions felt in class (Q1): The aim of this question was to explore teachers’ beliefs about the origin of the emotions experienced by students in the classroom (do they attribute emotions to external factors to the teacher or to the teacher him/herself?). The instructions were “Here are some emotions that pupils may experience in class. In your opinion, do they most often stem from the teacher’s practices or from other sources?” Respondents were asked to rate each of the nine emotions presented above on a 5-point Likert scale (1 = this emotion always comes from external factors to the teacher, 5 = this emotion always comes from the teacher). These items were developed specifically for this study to capture teachers’ direct attributions about the origin of each emotion. Because each attribution was assessed with a single item per emotion and the construct was conceptualized as emotion-specific rather than latent, internal consistency metrics are not applicable.
- Beliefs about the utility of emotion generated and experienced in class (Q2 + Q3): The respondent assessed to what extent they think the nine emotions described above, and which we specified were caused by classroom events, are useful to five different domains: learning, engagement, performance, relationship to the teacher, and relationship to the peers (five questions per emotions). The first three categories (learning, engagement, performance) were then averaged as they all relate to learning processes (see the data analysis strategy below). For example, the instructions of utility perceived for engagement were “The following questions focus on your perceptions of the different emotions that your (future) pupils may experience in relation to learning activities carried out in class: When a pupil feels [emotion] about the learning activity in class, it facilitates their engagement in the task.” Respondents were asked to answer on a 4-point Likert scale (1 = strongly disagree, 4 = completely agree). The items assessing perceived utility were developed for this study, drawing on prior work on academic emotions and their functions in learning (Fischer & Philippot, 2022; Reinhard Pekrun et al., 2002a, b). Each emotion was rated separately across five educational domains, consistent with our intention to capture differentiated, emotion-specific appraisals. Aggregated domain scores showed high internal consistency (*α* =.90), single emotion–utility items are not amenable to internal consistency estimates. This structure was designed to capture teachers’ intuitive and differentiated appraisals of emotional utility across various educational domains, without assuming an underlying latent structure across items or domains.
- Beliefs about the controllability of students’ emotions (Q3): Four questions were asked, adapted from the French version of Implicit Emotion theory scale (Congard et al., 2022; Tamir et al., 2007) on the malleability of emotions (“No matter how hard they try, pupils can’t truly change the emotions that they have”; “Every pupils can learn to control his/her emotions”; “The truth is, pupils have very little control over their emotions”; “If they want to, pupils can change the emotions that they have”). Cronbach’s alpha was 0.70 in our sample. Respondents were asked to answer on a 5-point Likert scale (1 = strongly disagree, 5 = completely agree).

#### Qualitative measures of teachers’ beliefs about emotions

Three open-ended questions were included for answering Q4 and Q5. For these questions, participants had to answer, with their own words: What do you think an emotion is? What role does it play in learning, according to you? What role does it play in the pedagogical relationship, according to you?

#### Description of larger project

The present work focuses on characterizing the beliefs that teachers in training have about emotions in the classroom and how they think these emotions impact learning processes and classroom relationships. This is however a question that is part of a larger project on the impact of individual differences between teachers, and how these may be associated with their vision of emotion, their teaching, and their beliefs about emotions in the classroom. As part of this larger project, five additional measures were gathered during the testing, but not analyzed further in the present work. These additional measures are:

- Emotional skills: We used the short French version of the PEC (Mikolajczak et al., 2014) to assess respondents' emotional skills via 20 items (intra- and interpersonal skills), evaluated with a 5-point Likert scale (Mayer & Salovey, 1997; Mikolajczak et al., 2014).
- Interpersonal regulation skills: A final closed question, formulated by us, explores teachers’ beliefs about their ability to help pupils manage their emotions in the classroom.
- Vision of emotion: With regard to qualitative measures, we did not analyze the answers to the question “What do you think an emotion is?” in order to focus more specifically on beliefs about the utility of emotion in learning processes and in the teaching relationship, which are also measured by quantitative measures. This gave us complementary data on the same questions. The open question not analyzed in this study will be dealt with in a future article.
- Vision of teaching: We used the French scale about teachers’ beliefs on learning and teaching to explore if they have a more traditional or socio-constructivist view of learning and teaching (Teaching and learning conceptions questionnaire; Chan & Elliott, 2004).
- Beliefs about the utility of emotion experienced in class but originated outside the class: This question was asked for externally originated anxiety/stress and anger, which are emotions that are very frequent in children (Johnco et al., 2015; Vierhaus & Lohaus, 2009) but that may not be exclusively related to classroom setup.

### Analytic strategies

#### Quantitative analysis

Data were averaged across participants. The main variables considered were the source of the nine considered emotions (from 1 = emotion always coming from factors that are extraneous to the teacher to 5 = emotions always coming from the teacher) and whether participants agree that each of the nine emotions is useful for a given domain (from 1 = totally disagree to 5 = totally agree). Three domains constitute this variable: utility for learning processes (which is the average between utility for learning, utility for engagement, and utility for performance), utility to the relationship with peers, and utility to the relationship with the teachers. The last variable consisted of the teachers’ belief in the malleability of emotions.

To examine if the perceived source of the emotions felt in class was differentially attributed depending on the emotion considered, we performed a repeated-measures ANOVA with one factor (the emotion, 9 levels) on the source of emotions variable. We a priori decided to focus on contrasting emotional categories of adjacent rank (8 comparisons), which permits to reduce the burden of multiple comparisons, yet rendering the analyses interpretable. Tukey’s post-hoc values of the eight comparisons were examined for this purpose.

To examine whether different emotions were perceived as differently useful for either learning processes or relationships in class, we conducted a repeated-measures ANOVA (with two within-subject factors: emotion and utility domain). We first examined in detail the main effect of emotions, to investigate whether future teachers consider some emotions more useful than others in class (irrespective of the “utility for what?” question). Tukey post hoc tests were conducted to answer this question. Then, we examined if different domains for utilities could interact with emotions, meaning that teacher would evaluate differently the utilities of different emotions depending on the utility domain (learning processes, relationship with peers, or relationship with the teacher). For this, we focused on the interpretation of the interaction effect given by the above-mentioned repeated-measures ANOVA. Tukey post-hoc tests were again conducted for contrasting emotions within a domain.

Finally, to test whether beliefs in the malleability of student’s emotions are associated with the way future teachers see the various utilities of emotion (independently of emotion types), we conducted linear regression analysis with two predictors: degree of beliefs in the malleability of the students’ emotions and domain of utilities.

### Qualitative analysis

We conducted a content analysis on the two open-ended questions selected for the present report. All quantitative and qualitative data were collected at the same time. After the quantitative analyses had been performed, only a subset of the open-ended questions (Q4 and Q5) was analyzed, in order to prioritize the qualitative material that most directly illuminated the quantitative findings, namely teachers’ beliefs about the utility of emotions for learning and for the teacher–student relationship. Other qualitative questions addressed broader or more conceptual aspects of emotion and were therefore not included in the present article. We used a qualitative approach inspired by thematic content analysis (Braun & Clarke, 2006). The analyzed responses addressed the two following questions (related to Q4 and Q5 described above):

Q4: What role do you think emotions play in learning?

Q5: What role do you think emotions play in the relationship with pupils?

First, all answers to each question were compiled into a single document. Two researchers of the team—accustomed to and trained in qualitative analysis in the field of emotions (Fischer et al., 2021)—read the entire corpus of data (292 responses) for an initial “floating reading” to become immersed in the data. The coding stage was subsequently carried out independently by both researchers. This coding process identified the main themes emerging from the data, transforming raw data into higher-level thematic categories (Yin, 2015). These categories emerged directly from the data.

Since a single response could contain multiple ideas related to one or different thematic categories, each new idea was referenced by a letter, and a number identified the respondent, allowing illustrative verbatim to be retrieved (e.g., 14A-14B-14C indicates that three different ideas are found in the response of respondent 14).

Each researcher generated a coding grid for both questions, which were then compared for allowing data triangulation. Points of disagreement were discussed, and final coding grids were created, comprising eight thematic categories for Q4 and six for Q5. Each category includes references to illustrative verbatim within the data (not included in the following results but available on request).

## Results

### Quantitative results

#### Perceived source of the emotions felt in class

**Fig. 1:**
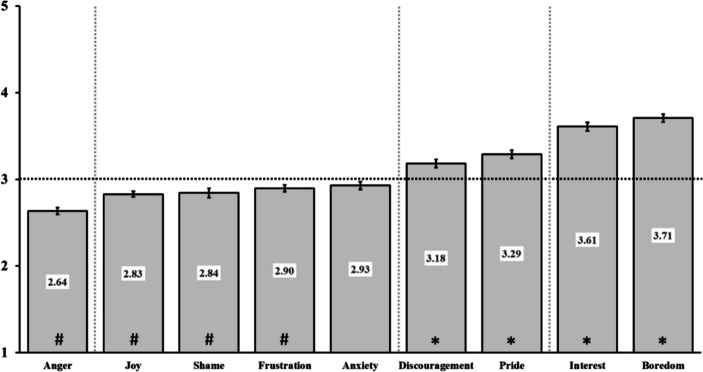
*Extent to which the teacher was considered the source of different emotions.* Vertical axis shows to what extent participants believed the teacher was the source of the different emotions a pupil can have in class (from 1 = totally external factors to 5 = teacher is the exclusive source of emotion). Error bars are standard errors of the mean. Vertical dashed lines indicate post-hoc differences between emotions, separating those whose levels are significantly different. Horizontal dashed line indicates the middle of the scale (emotions neither attributable to teacher, nor to external factors), # show bars that are significantly below this midline (attributable to external factor) while * show bars that are significantly above this midline (attributable to the teacher). These latter analyses are conducted via one sample *t*-tests with a mean of 3

To examine if the perceived source of the emotions felt in class was differentially attributed depending on the emotion considered, we performed a repeated-measures ANOVA with one factor (the emotion, 9 levels). Results showed a significant effect of the type of emotion, *F*_(8,2096)_ = 82.6, *p* <.001, *η*^2^ =.19 (see Fig. 1). Tukey ordinal post-hoc tests between each consecutive ranked emotions revealed four different groups of emotions. More specifically, they showed that emotions attributed mostly to the teacher were primarily Interest and Boredom (grouped mean = 3.66, SD = 0.58, *p* <.001 with middle value of attribution), followed (*p* *<.001*) by Discouragement and Pride (grouped mean = 3.24, SD = 0.58, *p* < *.001* with middle value of attribution). The third group of emotions (which different from the previous ones, all *p* < *.*001), included Anxiety, which had the particularity of its source being neither attributed to the teacher nor to external factors (or attributed equally to both, mean = 2.93, SD = 0.76, *p* =.12 with middle value of attribution). Anxiety was however not significantly different from the level shown for Frustration, Shame, and Joy. These last three emotions were significantly more attributed to external factors than to the teacher (mean = 2.86, SD = 0.42, *p* <.001 to *p* =.009 with middle value of attribution). Finally, Anger was the emotion that was the most associated with external factors of all the considered emotions (mean = 2.64; SD = 0.62, *p* <.001 with middle value of attribution). Participants judged this emotion has having the lowest probability of being due to the teacher than any other emotion (*p* <.001 to *p* =.004).

#### Perceived utility of the emotion felt in class

**Fig. 2:**
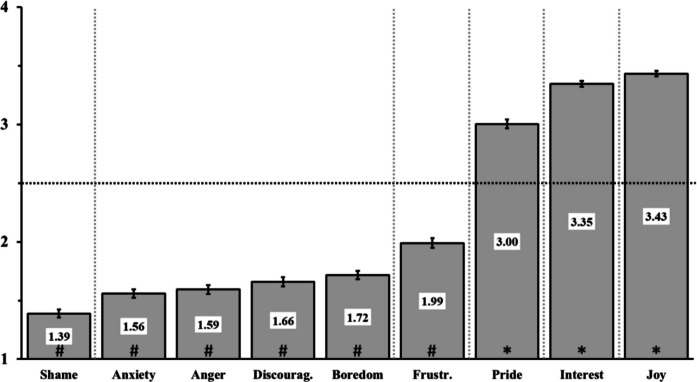
*Differential perceived utility of emotions for the learning and relationship in class.* Level of perceived utilities the different emotions could have, regardless of the type of utilities (learning or relationships). Scale goes from 1 = not useful to 4 = extremely useful. Error bars are standard errors of the mean. Vertical dashed lines indicate post-hoc differences between emotions, separating those whose levels are significantly different. Horizontal dashed line indicates the middle of the scale (emotions neither useful nor not useful), # show bars that are significantly below this midline (not useful) while * show bars that are significantly above this midline (useful for learning process). These latter analyses are conducted via one-sample *t*-tests with a mean of 2.5

We were then interested in whether different emotions were perceived as differently useful for either learning processes or relationships in class. A repeated-measures ANOVA (2 factors: emotion and utility domain) indeed showed a main effect of the emotion category, *F*_(8,2096)_ = 719.32, *p* <.001, *η*^2^ =.56 (see Fig. 2). Post-hoc tests showed that Joy was the emotion considered the most useful for learning, followed by Interest and Pride, with both relatively significant lower values. Interestingly, these are the three positive emotions of our pool. Then, came frustration, with a particular position: considered as not particularly useful, it however ranks higher than the other negative emotions, all considered equal in terms of utility (Anxiety, Anger, Discouragement, and Boredom). Finally, the emotion of Shame came out last in the ranking with a lower perceived utility as compared to all other emotions (mean score = 1.39; SD = 0.57, *p* <.001 with all other emotions) (Fig. 2).

**Fig. 3:**
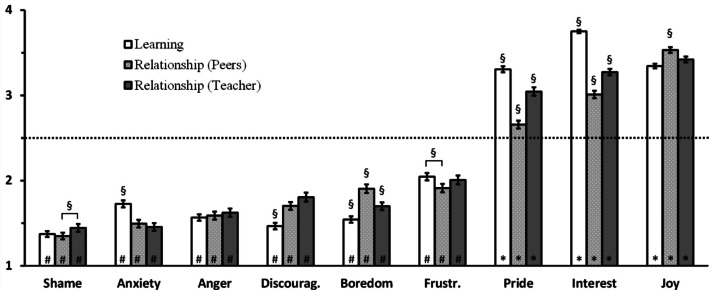
*Perceived utility of emotions depending on the utility domain (learning or relationships)*. Level of perceived utilities the different emotions could have, depending on the utility domain (for learning processes, white bars, relationship with peers, light gray dashed bars, or relationship with teacher, dark gray bars). Scale goes from 1 = not useful to 4 = extremely useful. Error bars are standard errors of the mean. Horizontal dashed line indicates the middle of the scale (emotions neither useful nor not useful), # show bars that are significantly below this midline (not useful), while * show bars that are significantly above this midline (useful for learning process). These latter analyses are conducted via one sample *t*-tests with a mean of 2.5. § show contrasts between utility domain level within each emotion

Then, we wondered if different domains for utilities could interact with emotions, meaning that the teacher would evaluate differently the utilities of different emotions depending on the utility domain (education domain, relationship with peers, or relationship with the teacher). This is indeed evidenced by a significant Emotion × Domain interaction shown by the Anova, *F*_(16,4192)_ = 52.71, *p* <.001, η^2^ =.05 (see Fig. 3). In terms of utility ranking, the most important emotion for *learning* was Interest, followed by Joy and Pride, which have similar utilities; then came Frustration, which, as for the previous results, was the first to be on the non-useful side. Interestingly, for learning, Anxiety had a special ranking, less useful than Frustration but more useful than the other four last “non-useful” emotions, i.e., Anger, Boredom, Discouragement, and Shame. Regarding the utility for the *relationship*, there were differences whether it was with respect to peers or to the teacher. Both had the same top-tier three emotions: Joy, more useful than Interest, itself more useful than Pride. Again, below the line, the relationship with peers profited similarly from Frustration and Boredom, followed by Discouragement, Anger, and Anxiety, with Shame being the least useful, significantly less than any other. On the other hand, the relationship with the teacher did not benefit from the last five negative emotions (Discouragement, Boredom, Anger, Anxiety, and Shame) and, this, in a similar fashion.

#### Perceived malleability of the emotion felt in class

**Fig. 4:**
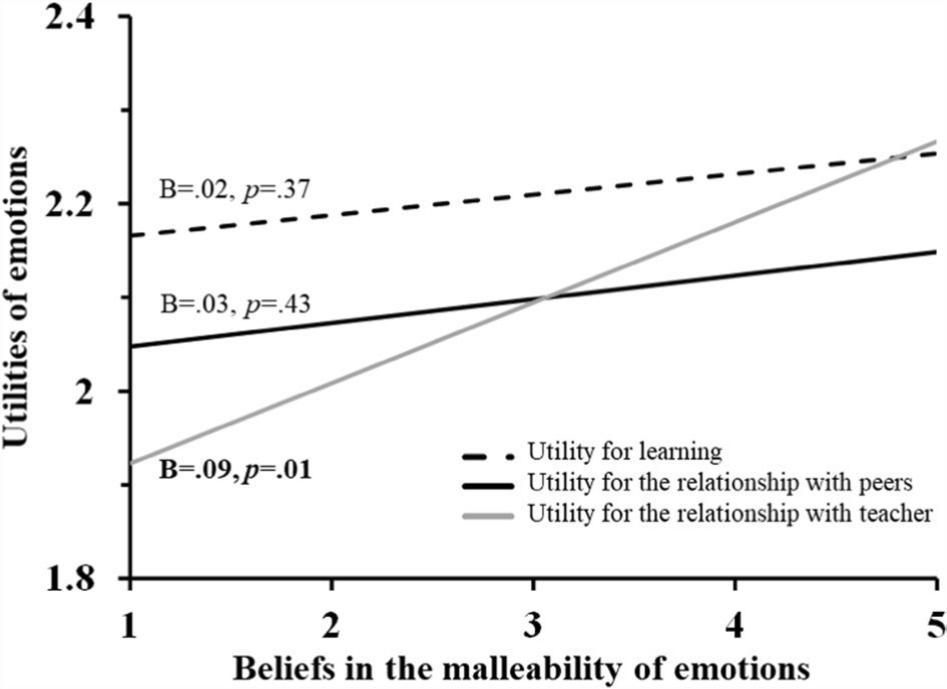
*Malleability beliefs relationship with the perceived utility of emotions for the different utility domains*. Levels of perceived utilities the different emotions could have, depending on the utility domains (for learning processes, dashed black line, relationship with peers, continuous black line, or relationship with teacher, gray line). Scale goes from 1 = not useful to 4 = extremely useful. Horizontal axis shows beliefs about the malleability of emotions. Slopes are indicated with B, and their significance

Finally, we tested whether the beliefs in the malleability of emotions are associated with the way people rank the various utilities of emotions (independently of emotion types). Results showed that this is the case for the extent to which the teacher thinks his/her emotions are malleable, *F*_(2,522)_ = 4.49, *p* =.012, *η*^2^ =.001. This result is illustrated in Fig. 4. Note that the main effect of Malleability was not significant, *F*_(1,261)_ = 3.21, *p* =.07.

### Qualitative results

#### Beliefs about the role of emotions in learning

Although we did not specifically address their source, utility, or malleability in the open-ended questions, some responses offered hints about the emotion perceived utility, particularly for so-called positive and negative emotions and are consistent with quantitative results. Few specific emotions were mentioned, giving a more holistic view of teachers’ beliefs about this subject compared to the closed items.

The analysis of the open-ended responses to the online questionnaire showed that respondents think that emotions play a role in learning, with many ideas describing this role as “important,” “essential,” “predominant.” The idea of not knowing whether emotions play a role in learning appeared only in very few responses (see Table 1 below with coding categories).

**Table 1:** *Thematic categories emerging from the data coded in question 4*

Thematic categories for Q4	Frequency of appearance of the category in the whole corpus	Quote example
1.No answer/incomplete answer that is difficult to interpret	23 times	“They can be used according to the situation”
2.The respondent indicates an inability to answer	3 times	“I don’t really understand the role of emotions in learning.”
3. The respondent acknowledges that emotions play a role in learning but does not identify a specific aspect or a particular mechanism of this influence	59 times	“It is impossible to separate emotion and learning”
4. The respondent indicates that emotions play an important role in learning and specifies the aspects they are involved in	- The respondent indicates that emotions play an important role in general (without distinguishing between positive and negative emotions): this idea appeared 129 times - The respondent specifies the negative role of “negative” emotions: this idea appeared 30 times: this idea appeared 39 times - The respondent specifies the positive role of “positive” emotions: this idea appeared 65 times - The respondent specifies the positive role of “negative” emotions: this idea appeared 3 times	“Emotions are important during learning because they play a key role in peer relationships, but also in the student’s ability to learn and the teacher’s ability to teach”
5. Because emotions play an important role in learning, the teacher must encourage them to emerge	2 times	“A student who is completely neutral in a learning situation is likely to have less interest in the subject matter and will therefore retain less of their experience of the situation and thus of the content at the ‘subject’ level. To maintain students’ attention and interest, it is therefore important to take the emotional aspect into account and, to a certain extent, try to elicit emotions in certain learning sequences.”
6. Emotions need to be decoded: identifying one’s emotions (or those of your students) is an important factor in learning	35 times	“Seeking to understand pupils’ emotions can help them overcome significant obstacles in their learning”
7. Emotions need to be regulated:	26 times	“Managing one’s emotions, accepting them, naming them and finding appropriate responses to different situations therefore seem to me to be objectives to work on at school”
8. Since emotions are important, they need to be regulated, and teacher should help pupils to achieve this regulation	3 times	“During school activities, children experience a range of emotions. They must learn to manage them, and the teacher is there to help them”

Some of the comments made by respondents remain general: many responses lacked clarity about the mechanisms through which emotions influence learning; “positive” emotions were seen as beneficial (sometimes with the explicit belief that only positive emotions can promote learning) and “negative” emotions were viewed as hindering learning, which is fairly consistent with the ANOVA results shown above. The positive role for a “negative” or unpleasant emotion was mentioned only 3 times.

Other responses provided interesting information about the effects of emotions on different dimensions of learning. The most frequently reported impact, whether discussing general effects of emotions or specific impacts of positive and negative emotions, was on motivation. The effects of emotion on cognition appeared next but in a different order for “negative” and “positive” emotions. According to respondents, “negative” emotions tended to distract students’ attention from learning and impede optimal learning conditions, while “positive” emotions influenced how students perceived new knowledge.

In terms of teacher training, categories 6 to 8 were particularly noteworthy. These responses indicated that future teachers believe emotions play a crucial role in the classroom and need to be identified and decoded. They saw emotions as signals that should be taken into account to enhance self-understanding and learning (category 6). In addition, respondents indicated that emotions also need to be regulated, controlled, or contained to ensure effective learning (category 7). Some respondents felt it is part of a teacher’s role to support students in this regulation (category 8, although this view is less common).

### Beliefs about the role of emotions in teacher-student relationship

As a preliminary point, it should be noted that we chose to investigate, in this study, teachers’ beliefs about relationships with students (and not relationships between peers). We made this choice following analysis of the quantitative data, which in Fig. 4 shows that the link between beliefs about malleability and utility of emotions is more present in the relationship with the teacher than with peers. We present the results of Q5 using the same structure as for the previous question.

Categories 1 and 2 were sparsely supplied, indicating that few respondents provided no answers, uninterpretable answers, or indicated an inability to answer.

The following categories highlighted the different beliefs about the role of emotions in the student–teacher relationship, ranging from the most general to the most specific theories (Table 2).

**Table 2:** *Thematic categories emerging from the data coded in question 5*

Thematic categories for Q5	Frequency of appearance of the category in the whole corpus	Quote example
1.No answer/incomplete answer that is difficult to interpret	15 times	“It’s something we have in common.”
2.The respondent indicates an inability to answer	2 times	“I don’t know”
3. The respondent think that emotions should not play a role in this relationship	1 time	“Emotions should not play a role”
4. The respondent states in general terms that emotions play a role in the classroom (but without specifying the student–teacher relationship and recalling elements linked to Q4)	22 times	“The role of emotions is crucial because learning is linked to how the student feels”
5. The respondent believe that emotions play a role in the educational relationship but does not identify a specific aspect, the nature of the relationship, or a particular mechanism of influence	67 times	“Importantly, we are all capable of emotions, with differences between individuals. These emotional factors must be taken into account in relationships with students.”
6. The respondent indicates that emotions play an important role in the teacher-student relationship and specifies which variables they affect	The respondent expressed the idea of the a) impact of emotions on classroom climate: this idea appeared 138 times b) impact on the student: this idea appeared 133 times c) impact on the teacher: this idea appeared 59 times	a)”Teachers must facilitate their learning ability by creating a climate of trust and security” b) “We must show the child that we hear their emotions and pay attention to them.” c) “Teachers’ emotions in teacher-student relationship can help teacher to better understand certain situations, but they can also hinder the effectiveness of teaching (if teacher can’t manage their emotions)”

First, this thematic content analysis highlighted that all the future teachers questioned (with one exception) considered emotions to play a role in their relationships with students. Some minority responses (category 4) referred to the role of emotions in student learning and teaching in general, thus overlapping with question 4. Nonetheless, the future teachers reported very rich beliefs about the role of emotions in these relationships and their impact on students, teachers, and the classroom climate.

Category 5 presents beliefs recognizing the importance of emotions in this relationship. Respondents either limited themselves to this general idea or further qualified or justified their views. They explained that emotions can regulate teacher-student interactions, act as a “bridge” connecting teachers and students in a universally experienced phenomenon, serve as indicators of the quality of the pedagogical relationship (a highly represented view), and influence teaching practices. Some noted that while emotions play a role in relationships with students, the teacher must remain a professional, suggesting that considering emotions might lead teachers to lose objectivity or deviate from their role (being a psychologist and not a teacher).

Category 6 includes beliefs which recognize that a relationship with a student cannot ignore reciprocal emotions impacting various school context variables. These data are organized on 3 dimensions: (A) impact of emotions on general classroom climate; (B) on the student, and (C) on the teacher.

A) We present the data that showed beliefs about the influence of emotions on relationships and classroom climate. The idea that emotions help create a relationship of trust, security, and respect between teachers and students appeared 138 times.
B) The data highlighted some beliefs (appearing 133 times) about the influence of emotions on the students. Respondents indicated that a teacher who considers emotions in their relationship with students views them more as individuals, understanding their needs and helping them develop emotional skills. Respondents also perceived that this relationship has an impact on their learning: a good quality relationship improves learning, academic performance, motivation, interest, and the learner’s perception of the situation, and would also enable a better understanding of the teacher’s expectations. Conversely, a poor relationship can diminish a student’s desire to attend school and negatively influence their self-esteem, motivation, and performance. The data also suggested that, according to the questioned teachers, this relationship affects students’ personal development and well-being.
C) Respondents also reported that considering students’ emotions within the relationship allows the teacher to adapt their relationship or teaching practices, particularly their ability to empathize (reported 59 times). Other beliefs highlighted the role of the teacher’s own emotions in the student relationship. Taking into account one’s own emotions in relation to the student would sometimes be in the service of one’s teaching practices, but not always (the risk of lacking objectivity towards the pupils is also found here). The teacher’s ability to regulate emotions to teach effectively and build relationships was also reported in this category.

Categories 5 and 6 were particularly noteworthy for teacher training on the relational dimensions of the profession. Indeed, teacher training students say that emotions and relationships with students are interconnected; the relationship formed with the student has multiple influences on the school context (for the student, the class, and the teacher).

## Discussion

Our results about teachers’ implicit theories of emotions are fairly close to what the literature highlights about the general role of emotions in learning: unanimous recognition of the role of emotion in learning and in different cognitive processes (Brosch et al., 2013), generally positive effects of pleasant emotions and generally deleterious effects of unpleasant emotions (Pekrun, 1992), recognition of the role of emotion in student–teacher relationship (de Ruiter et al., 2021), seen as a place where a variety of emotions emerge, and conditioning the school climate and learning outcomes (Hofkens & Pianta, 2022).

However, future teachers’ implicit conceptions of their students’ emotions remain caricatured and fail to consider the potentially complex effects of emotions on cognition. Indeed, various research studies have highlighted the deleterious effect of pleasant emotions on learning under certain conditions, as well as the sometimes beneficial effect of unpleasant emotions on learning (Pekrun, 2006; Tan et al., 2021). In particular, we find that future teachers’ emotional beliefs about their students’ emotions are primarily concerned with the valence of emotions, without taking into account other emotional characteristics that have an impact on learning, such as the frequency or emotional arousal, recognized as a key factor in understanding the effect of an emotion on learning (Storbeck & Clore, 2008).

Concerning the results on future teachers’ beliefs about the source of emotions**,** they showed globally that future teachers believe Boredom and Interest are the two emotions they mostly triggered among their students, followed by Pride and Discouragement. This is a mix of negative and positive emotions. Interestingly, other emotions that may be important for improving performance (such as joy) are considered as coming essentially from external factors. The result showing that future teachers attribute joy predominantly to external factors might indicate a gap in teachers’ understanding of how intrinsic classroom elements contribute to student well-being and learning. This perception could hinder their ability to implement strategies that actively promote positive emotions and consequently miss opportunities for embedding joy-inducing elements, such as gamified learning or collaborative projects, directly into classroom practices, which were shown critical for performance and resilience in learning (Perez-Aranda et al., 2024). On the other side of the spectrum regarding emotion source, the case of interest is particularly noteworthy. Interest has been brought forward as a major epistemic emotion, fundamental to learning processes, particularly regarding motivational dynamics and engagement (O’Keefe et al., 2017). We think that teachers acknowledging their role in fostering student interest demonstrate an awareness of how specific pedagogical strategies enhance cognitive engagement. Instilling this belief in teachers' curricula is essential, as only educators who recognize their influence on students' interest are likely to design and implement practices that nurture and sustain it throughout their careers. Unlike joy, which is seen as arising from external or incidental factors, interest is more readily linked to teachers’ intentional instructional choices, making it easier for future educators to recognize their role in actively cultivating it. Future research could explore whether these attributions extend to other epistemic emotions, such as curiosity, which has been linked to socio-cognitive conflict and deeper learning (Ostroff, 2016).

The recognition that not only positive but also negative emotions could arise from teaching practice is very important. Indeed, teachers’ implicit theories about emotions could be an indicator of how they take them into account to process their practice (Ahn, 2005; Zammuner, 2023). We believe that thinking that negative emotions can arise in students due to their own behavior allows the teacher to reflect more broadly on his/her implemented practices and how these do or do not meet students’ learning needs (autonomy, competence, social connection; Niemiec & Ryan, 2009). Only careful consideration of the full valenced spectrum of the potentially triggered emotions can guide a practice implementation that would foster learning and well-being of the students in class.

Concerning the perceived utility of emotions, we found that our respondents believe that positive emotions were the most useful for class activities, whereas negative emotions are the less useful. Their implicit theories hence align with previous results showing in qualitative analysis and in literature (Reschly et al., 2008; Williams et al., 2013). At the other end of the spectrum, we find shame, which teachers-to-be identify as an emotion that can damage classroom dynamics by eroding trust and making students or teachers less willing to engage fully. They likely recognize that it could harm the social-emotional climate of the classroom. This aligns with our qualitative findings about the role of emotions in teacher-student relationships and with previous literature (Johnson, 2012). Shame, which could have been used historically as a way to motivate student learning and behavior to avoid social judgement, seems now totally discarded from the classroom.

Details per domain showed other interesting results about this question, revealing that Pride and Interest are more useful for learning than for relationships, according to our responders. On the contrary, emotions like Boredom foster relationship with peers more than another domain. This result is particularly interesting as Boredom is typically framed as a counterproductive emotion, linked to disengagement and reduced academic performance (see, e.g., Sharp et al., 2020). However, some other studies (Barbalet, 1999; Sandstrom & Rawn, 2015; van Tilburg & Igou, 2016) suggest that Boredom could drive social interactions as students seek stimulation through peers, often during unengaging tasks. The fact that boredom is less associated to learning may come from teachers associating boredom with distraction and reduced focus in academic contexts, while recognizing that the same drive for stimulation can create opportunities for interpersonal engagement. In other words, boredom is seen as a social signal rather than a learning resource. This highlights its social utility as a potential motivator for collaboration or bonding in the face of monotony. Teachers could hence foster collaborative problem-solving for less engaging activities, turning this emotion into an opportunity for peer connection.

Interestingly, frustration appears to be most important for learning, if it had to be useful for something. Traditionally seen as a negative emotion, frustration emerges now to play a possible productive role in learning. Research indeed suggests that moderate frustration, when coupled with effective scaffolding, fosters persistence, problem-solving, and deeper cognitive engagement (Baker et al., 2010; Tian et al., 2021). Our results encourage the emerging understanding of frustration as a signal for growth, rather than a purely negative experience.

Altogether, these results suggest that teacher education programs could emphasize strategies to balance the different roles of emotions, ensuring both cognitive and relational benefits in the classroom.

The last quantitative analysis tested whether the beliefs in the malleability of emotions are associated with the way people evaluate the various utilities of emotion. This was not confirmed at a general level, but further results showed that the association is present only when we consider the relationship with the teacher (and not when we consider learning or the relationship with peers). We could argue that future teachers who think it is possible to regulate emotions will recognize emotion as more useful to the relationship. This result highlights the context-sensitive nature of emotional beliefs and their impact on interpersonal dynamics. Incorporating modules that explore the malleability of emotions and the role in making the professionals appraise the emotions’ utility in classroom interactions can help prospective teachers recognize the context-specific benefits of emotional adaptability.

In practical perspectives, we support the idea that initial teacher training should provide up-to-date scientific insights into the role of “positive” and “negative” emotions in both learning and teaching. Notably, initial and continuous training may present teachers with theories addressing the emergence of students’ emotions in the classroom, such as the control-value theory (Pekrun & Goetz, 2023). This theory highlights pedagogical strategies enabling students to enhance control and values. Such training could lead teachers to avoid unintentionally perpetuating some misconceptions through ineffective teaching strategies or misinterpretations of educational psychology (McAfee & Hoffman, 2021) and help them to adopt practices improving quality learning and relational climate. In addition, this study encourages to consider, in future research, the wealth of teachers’ beliefs about students’ emotions and to better understand how these emotional beliefs could guide their effective teaching strategies.

This study has several limitations. Firstly, all the data are self-reported, which may induce social desirability biases in the data depending on what is culturally encoded as acceptable to say. Future research, taking an intercultural perspective, could explore how the social/cultural/professional contexts influence these beliefs. In addition, although we did not focus our attention on cultural differences in emotions’ beliefs between Belgian and Swiss teachers, it would be interesting to do so in a future study, given that we know culture shapes our emotions and beliefs (Decuir-Gunby & Williams-Johnson, 2008). Secondly, we did not compare the emotional beliefs of student teachers with those of experienced teachers. It would be very interesting if future research could explore these same beliefs in teachers with long teaching experience in order to assess the congruence/divergence of emotional beliefs in these two groups. Finally, it is likely that the order of the questions influenced the participants’ responses. A future study should be careful to vary the presentation of the questions to limit this bias.

## Data Availability

The data is available on request from the author (it’s still being partly analyzed for future publication).
